# Comparison of Endoscopic Vacuum Therapy and Endoscopic Stent Implantation With Self-Expandable Metal Stent in Treating Postsurgical Gastroesophageal Leakage

**DOI:** 10.1097/MD.0000000000003416

**Published:** 2016-04-22

**Authors:** Jae J. Hwang, Yeon S. Jeong, Young S. Park, Hyuk Yoon, Cheol M. Shin, Nayoung Kim, Dong H. Lee

**Affiliations:** From the Department of Internal medicine, Seoul National University College of Medicine, Seoul National University Bundang Hospital, Seongnam, Korea.

## Abstract

The aim of the present study was to evaluate the more effective therapy for the postsurgical gastroesophageal leakage by a head-to-head comparison of endoscopic vacuum therapy (EVT) and endoscopic stent implantation with self-expandable metal stent (E-SEMS).

In this hospital-based, retrospective, observative study, the patients were classified into 2 groups. Those treated with EVT were assigned to the EVT group (n = 7), and those treated with E-SMS were assigned to the E-SEMS group (n = 11). We evaluated the clinical characteristics and treatment outcomes between the 2 groups.

All 7 patients (100%) were treated with EVT, but only 7 of 11 patients (63.6%) in the stenting group were treated successfully. The median time to clinical success was 19.5 (5–21) days in the EVT group and 27.0 (3–84) days in the E-SEMS group. The median hospital stay was 37.1 (13–128) days in the EVT group and 87.3 (17–366) days in the E-SEMS group. The complicaion rate was lower in the EVT group (0/7, 0.0%) than that in the E-SEMS group (6/11, 54.5%) with statistically significant difference (*P* = 0.042).

EVT is more effective and has fewer adverse effects than E-SMS therapy as a treatment for postsurgical gastroesophageal leakage.

## INTRODUCTION

Postsurgical esophageal leakage is a serious complication. The reported incidence of esophageal leakage after esophagectomy or proximal gastrectomy ranges from 3% to 25%.^1–6^ A small esophageal leak may cause severe mediastinitis and sepsis and is associated with a mortality rate of 3% to 10%.^7^ Surgical treatments have traditionally been applied for postsurgical esophageal leakage, but re-operation is associated with mortality rates of 20% to 32%, even in specialized tertiary units.^8–10^ Over the past 10 years, as endoscopic techniques have developed, several endoscopic treatment options have been used to control postsurgical esophageal leakage, such as approximation with endoclipping, injection with fibrin glue or histoacryl tissue adhesive, or endoscopic implantation with self-expandable metal stent (SEMS) or self-expanding plastic stent (SEPS).^11–17^ However, approximation with endoclipping requires several procedures, especially in cases of large anastomotic defects, and an additional procedure for stent implantation. Injection of fibrin glue or histoacryl tissue adhesive is associated with a risk of thrombosis or embolization. Of the several endoscopic treatment procedures, endoscopic stent implantation has been considered more effective treatment for postsurgical esophageal leakage, with a clinical success rate of over 80%.^13,15–17^ However, several studies have reported that endoscopic stent implantation is associated with several problems such as stent migration, difficulty of stent removal owing to tissue growth, and stricture development after stent removal.^16,17–20^

Recently, endoscopic vacuum therapy (EVT) has been introduced as an effective treatment modality for postsurgical esophageal leakage.^21,22^ Endocavitary vacuum therapy with the use of a sponge system to close was first described by Weidenhagen for controlling anastomotic leakage in rectal surgery.^23^ Further this method was extended to the upper gastrointestinal tract theoretically in Germany, first reports were published.^21,22^ There were 3 previous studies available concerning comparison of EVT and stent and these studies are all in favor of EVT.^24–26^ However, in Asia, there have been no comparative studies between EVT and endoscopic stent implantation with self-expandable metal stent (E-SEMS).

The aim of the present study was to evaluate the more effective therapy for the postsurgical gastroesophageal leakage by a head-to-head comparison of EVT and E-SEMS.

## MATERIALS AND METHODS

### Study Population

This study was conducted at Seoul National University Bundang Hospital between January 2008 and December 2014. The medical records of 18 patients who had postsurgical esophageal leakage after esophagectomy or gastrectomy and were treated with EVT or E-SEMS were retrospectively reviewed. The patients were classified into 2 groups. Those treated with EVT were assigned to the EVT group (n = 7) and those treated with E-SMS were assigned to the E-SEMS group (n = 11). All patients gave written informed consent for procedures before treatment, and this study was approved by the Ethics Committee at Seoul National University Bundang Hospital. (IRB number: B-1501/282–109).

### EVT

**FIGURE 1:**
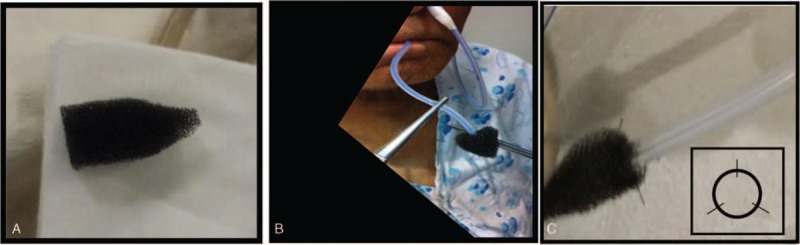
(A) Size-adjusted sponge with pore size of 400–600 μm. (B) A nasogastric tube inserted into one nostril after applying a lubricating gel, then extracted through the oral cavity with forceps. (C) Endoscopic vacuum therapy (EVT) sponge-fixed using an applied 3-way nylon suture to a nasogastric tube with the side-hole removed.

**FIGURE 2:**
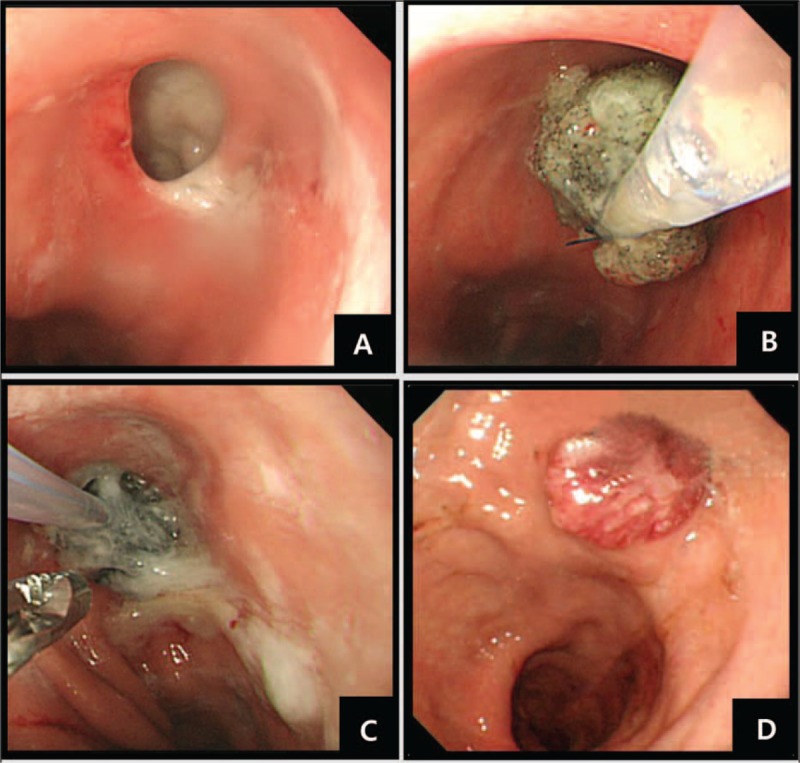
Endoscopic images of postsurgical esophageal leakage treated with endoscopic vacuum therapy (EVT). (A) A large defect observed by esophagoduodenoscopy. (B) Endoscopic placement of a drainage nasogastric tube armed with a size-adjusted sponge, which is applied using a 3-way nylon suture. (C) Sponges exchanged using alligator forceps. (D) Complete healing with no residual defect 60 days after EVT therapy.

Only 1 expert endoscopist (YSP) performed all endoscopic interventions. Before the sponge was inserted, endoscopic debridement of wound cavities was performed by using a regular biopsy forceps (FB-21K-1, Olympus, Japan). A nasogastric tube (Levin 16 French; Insung Medical, Seoul, Korea) was pushed into one nostril after applying a lubricating gel, and then extracted through the oral cavity with grasping forceps (FG-42L-1, Olympus, Japan). The side holes were removed from the nasogastric tube to maintain negative pressure. A size-adjusted (15–30 mm) polyurethane sponge of pore size 400 to 600 μm (KCI, San Antonio, TX) was sutured to the distal end of the nasogastric tube using a 3-way nylon suture (45 mm, Woorhi Medical, Seoul, Korea)(Figure 1A, B). The sponge was fashioned to the specific wound size as estimated by the endoscopist. The sponge size had to be smaller than the wound cavity to promote fistula collapse and closure. The sponge was grasped with a grasping forceps (FG-42L-1, Olympus, Japan) and introduced the necrotic cavities with a standard forward-viewing endoscope (Olympus H260; Olympus Optical, Tokyo, Japan) (intracavitary endoscopic vacuum method, Figure 1C). The open pore foam adheres to the tissue when connected with an electronic vacuum device (KCI V.A.C. Freedome®, KCI USA Inc., San Antonio, TX, setting: –125 mmHg, continuous, and high intensity). Representative results of the process are shown in Figure 2. According to recommendations for cutaneous VAC systems, the sponge was exchanged twice a week, until the grounds of the cavity appeared firmly closed.^27,28^ The nutrition of patients was a parenteral nutrition at the initiation of the EVT, switched to the enteral nutrition according to the healing state of the cavity.

### E-SEMS Therapy

In the E-SEMS group, 3 types of fully or partially covered SEMS were placed endoscopically. The types of stent utilized were a Niti-S stent (diameter of the shaft, 18 mm; diameter at the proximal throat, 26 mm; Taewoong Medical, Seoul, Korea), Hanaro stent (diameter of the shaft, 22 mm; diameter at the proximal throat, 30 mm; M.I.Tech, Pyeongtaek, Korea), or Bona stent (diameter of the shaft, 20 mm; diameter at the proximal throat, 28 mm; Sewoon Medical, Cheonan, Korea). The stents were removed within 4 to 6 weeks, before tissue growing around them. Endoscopic stent insertion was performed 1 to 4 times. Complications of E-SEMS were defined as bleeding, perforation, stent migration, difficulty of stent removal owing to tissue growth, and stricture development after stent removal.^16,17–20^

### Assessment of Treatment Outcome

The primary outcome evaluated was the clinical success rate. Clinical success in the E-SEMS group was defined as complete healing of the perforation or leakage by placement of a single or multiple stents irrespective of whether the stent was left in situ or was removed. Clinical success in the EVT group was defined as complete healing of the perforation or leakage by EVT irrespective of whether multiple endoscopic vacuum therapies were utilized. Clinical failure was defined as persistent leakage at follow-up, surgical resection for persistent leakage, or death before complete healing. Complete healing was confirmed by esophagography and esophagogastroduodenoscopy. The secondary outcomes evaluated were time to clinical success, duration of hospital stay, and complication rate.

### Statistical Analysis

Statistical analysis was performed using SPSS software package version 22.0 (Statistical Package for the Social Science, IBM Corporation, Armonk, NY). Data analysis was performed to evaluate the primary and secondary outcomes. After therapy, to assess the treatment outcome with EVT or E-SEMS, age, sex, distance of defect from upper incisor, defect size, number of sponge or stent exchange, clinical success rate, time to clinical success, total hospital day, and complication rate were analyzed. Student *t* test was used to evaluate continuous variables, whereas Pearson *χ*^2^ test and Fisher exact test were used to assess noncontinuous variables. *P* values <0.05 were defined as statistically significant.

## RESULTS

### Characteristics of Patients

A total of 18 subjects treated for postsurgical gastroesophageal leakage (14 male and 4 female) were analyzed in this study. Patient characteristics are shown in Table 1. The ages of the all patients ranged from 55 to 81 years. Of the 18 patients, 7 were in the EVT group and 11 were in the E-SEMS group. In the EVT group, 5 patients had undergone Ivor-Lewis for esophageal cancer, 1 patient had undergone total gastrectomy for advanced gastric cancer, and 1 patient had undergone segmental esophageal resection for gastrointestinal stromal tumor (Table 1). In the E-SEMS group, 3 patients had undergone Ivor-Lewis for esophageal cancer, 5 patients had undergone total gastrectomy for esophageal, early or advanced gastric cancer, 2 patients had undergone proximal subtotal gastrectomy for early gastric cancer, and 1 patient had undergone Mckeown esophagectomy for esophageal cancer (Table 1). The insufficiency in Case 9 and 16 was located above the upper esophageal sphincter after proximal subtotal gastrectomy, at 35 cm from the incisors in Case 9 and at 37 cm from the incisors in Case 16.

**TABLE 1:**
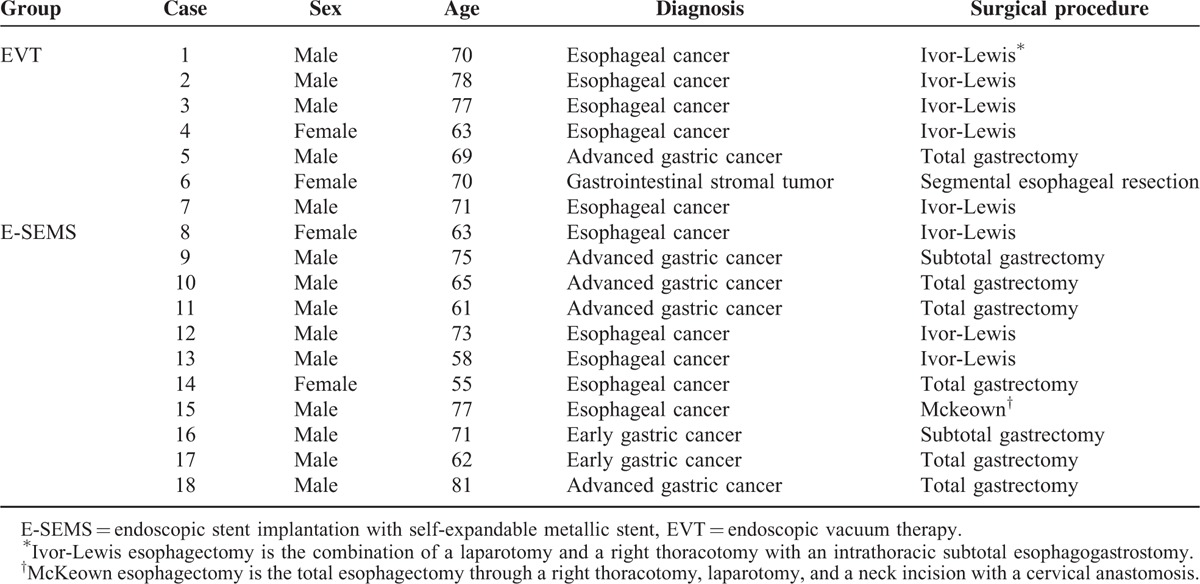
Characteristics of the 18 Patients Treated for Postsurgical Gastroesophageal Leakage

### Clinical Results and Outcomes

Clinical results and outcomes of the patients treated with EVT or E-SEMS are shown in Table 2. The median age was 71.1 (63–78) years in the EVT group and 67.3 (55–81) years in the E-SEMS group (*P* = 0.254). There were no differences in the male-to-female ratios, distance of defect from upper incisor, defect size, and number of sponge or stent exchange between the 2 groups (*P* > 0.05). All 7 patients in the EVT group were initially treated successfully. One patient showed recurrence on follow-up esophagogastroduodenoscopy and esophagography, but this was successfully treated with repeat EVT. The clinical success rate was higher in the EVT group (7/7, 100.0%) than that in the E-SEMS group (7/11, 63.6%), but there was no significant difference (*P* = 0.351). The median time to clinical success and median hospital stay were shorter in the EVT group than in the E-SEMS group, but there was no significant difference. However, the complication rate was lower in the EVT group (0/7, 0.0%) than that in the E-SEMS group (6/11, 54.5%) with statistically significant difference (*P* = 0.042).

**TABLE 2:**
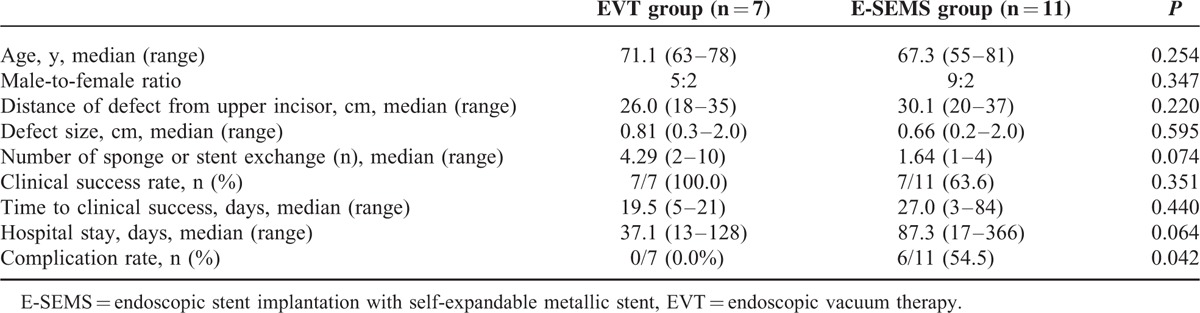
Clinical Results and Outcomes of Patients Treated With EVT or E-SEMS for Postsurgical Gastroesophageal Leakage

### Treatment Failure and Complications of the E-SEMS Therapy

In this study, 4 of 11 cases in the E-SEMS group were considered clinical failures. The remaining 4 patients had persistent leakage. One of 4 patients with clinical failure changed the treatment course to EVT therapy and was then successfully treated. The other 3 of 4 patients with clinical failure changed the treatment course to surgery, but 2 of 3 patients died of cancer progression before complete healing. Moreover, there were 6 cases of complication (3 cases of stent migration and 3 cases of difficulty of stent removal owing to tissue growth). In one case of stent migration, we tried to reposition and re-stent, finally failed, and changed to EVT. In the other 2 cases of stent migration, we succeeded with stent repositioning. In 3 of the 7 patients who were successfully treated with E-SEMS, the SEMS could not be removed because of tissue growth. In 4 of the 7 patients who were successfully treated with E-SEMS, the stents were removed successfully; the median closure time was 14 days (4–30 days).

## DISCUSSION

The results of our study showed that the clinical success rate in the EVT group was higher than that in the E-SEMS group (100.0% vs 63.6%), although this feature did not reach statistical significance. However, the complication rate in the EVT group was lower than the adverse event rate in the E-SEMS group with statistically significant difference (0.0% vs 54.5%, *P* = 0.042).

In our review of the published data of EVT for the management of upper gastrointestinal defects recent 2 years, EVT showed a high clinical success rate similar to our study (86%–100%, Table 3).^26,29–32^ The median number of sponge exchange and time to clinical success in our study were also similar to the median of other EVT studies (4.29 vs 4.77, 19.5 vs 17.8 days, Table 3).^26,29–32^ Loske et al^30^ showed a clinical success rate of 100% (10/10) for the iatrogenic esophageal perforations with intraluminal or intracavitary EVT. All perforations were healed in within a median of 3 to 7 days. Moreover, there was no stenosis, complication, and additional operative treatment. Kuehn et al^32^ found that a successful EVT for upper gastrointestinal defects with local control of the septic focus was achieved in 19 of 21 patients (90.5 %). The median number of sponge exchange was 5 (range, 1–14) and median time of therapy was 15 days (range, 3–46 days).

**TABLE 3:**
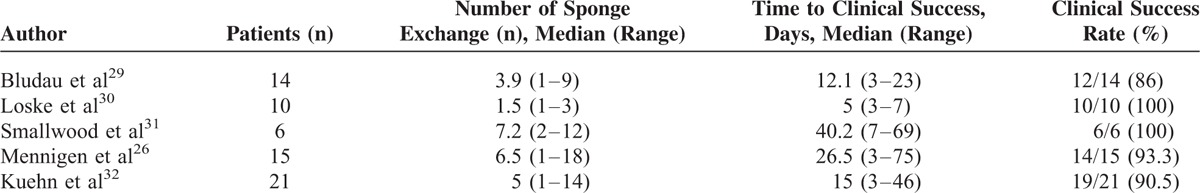
Published Data of Endoscopic Vacuum Therapy for the Management of Upper Gastrointestinal Defects Recent 2 Years (n = 66)

The comparison between EVT and stent implantation for the management of upper gastrointestinal defects, the published 3 studies reported that EVT showed higher clinical success rate and lower mortality rate compared with stent implantation (Table 4).^24–26^ The clinical success rate of EVT and stent implantation in our study was higher compared with the median of other studies (100.0% vs 87.5%, 63.6% vs 54.6%, Table 4). The median time to clinical success of EVT and stent implantation in our study was shorter than that of other studies (19.5% vs 41.4%, 27.0% vs 38.8%, Table 4). Brangewitz et al^25^ analyzed the outcomes of 32 patients treated with EVT and 39 patients with stent placement for esophageal leaks. The overall closure rate was significantly higher in the EVT group (84.4%) compared with the Stent group (53.8%). Mennigen et al,^26^ in a study on 45 patients with anastomotic leak after esophagectomy, compared the efficacy of EVT and stent placement. The 7 patients of the stent group switched to the EVT group during the therapy because of failure of stent therapy. The success rates were 86.4% for the EVT group and 60.9% for stent therapy by final therapy, but there was no significant difference.

**TABLE 4:**
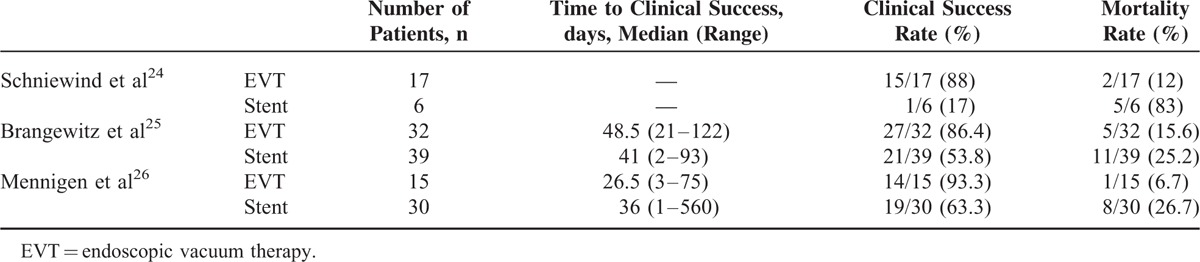
Published Data of Comparison of EVT and Stent Implantation for the Management of Upper Gastrointestinal Defects (n = 139)

EVT with persistent negative pressure makes rapid removal of necrotic debris or pus possible, and prevents further spread of contamination. The continuous suction promotes tissue granulation by reducing interstitial edema. It is also possible to close a postsurgical esophageal defect.^27,33^ Furthermore, EVT can be used in a grossly intraluminal wound. EVT has no serious complications compared with E-SEMS.^28,34^ In our study, there was also no serious complication in the EVT group compared with E-SEMS group. Therefore, EVT might be considered a better and more physiologic treatment modality than E-SEMS for postsurgical gastroesophageal leakage.

For the different variants of EVT, intracavitary and intraluminal techniques have been developed.^27^ The sponge drainage system is placed through an intestinal defect into an extraluminal wound cavity in the intracavitary EVT, the sponge is placed directly onto the defect within the lumen of the digestive tract in the intraluminal EVT.^27^ In our study, all patients were treated with intracavitary therapy method. Most of the investigators in previous studies first placed the drainage (with sutured foam) orally with grasper along an overtube. After correct placement, drainage tube was changed from orally to nosily. However, we used a modified method for placing of the polyurethane drainage. We pushed a nasogastric tube into one nostril after applying a lubricating gel, and then extracted through the oral cavity with grasping forceps. A size-adjusted polyurethane sponge was sutured to the distal end of the nasogastric tube. The sponge was grasped with a grasping forceps and introduced the necrotic cavities with a standard forward-viewing endoscope. The EVT procedure was introduced in our clinic in 2008 by literature. In the process of administering EVT to our patients, we modified the placing procedure of the polyurethane drainage to suit our condition.

This study has some limitations. First, this study enrolled a small number of cases; therefore, it is difficult to compare the outcomes between EVT and E-SEMS group with statistically significant difference. Second, this study is retrospective and single-center design study. No statistically significant results were generated from the comparisons made in this study. Third, the clinical treatment success rate of E-SEMS was lower compared with the previous studies.^13,15–17^ In our study, 2 patients in the E-SEMS group were died of cancer progression before complete healing. Although E-SMES was performed successfully, the 2 patients were poor candidates and very rapid cancer progression occurred in the patients.

In conclusion, EVT is more effective and has fewer adverse effects than E-SEMS therapy as a treatment for postsurgical gastroesophageal leakage. We believe that the advantages of EVT include a shorter median closure time, shorter hospital stay, and lower complications compared with E-SEMS therapy, which make it a potential alternative option for treating postsurgical gastroesophageal leakage. Further large-scale prospective studies are required to determine the broad application of this therapy in comparison with currently approved E-SEMS therapies.
